# Evaluation of time-dependent phenotypes of myocardial ischemia-reperfusion in mice

**DOI:** 10.18632/aging.205103

**Published:** 2023-10-10

**Authors:** Xiang-Min Meng, Jing-Han Yuan, Zhen-Fang Zhou, Qi-Pu Feng, Bing-Mei Zhu

**Affiliations:** 1 Regenerative Medicine Research Center, West China Hospital, Sichuan University, Chengdu, Sichuan, China; 2 Animal Experiment Center, West China Hospital, Sichuan University, Chengdu, Sichuan, China

**Keywords:** myocardial ischemia-reperfusion, myocardial apoptosis, inflammation, cardiac fibrosis, heart failure

## Abstract

Background: A mouse model of myocardial ischemia-reperfusion (I/R) is widely used to study myocardial ischemia-reperfusion injury (I/RI). However, few studies focus on the direct comparison of the extent of pathological events resulting from variant durations of ischemia and reperfusion process.

Methods: A mouse model of I/RI was established by ligation and perfusion of the left anterior descending coronary artery (LAD), and the dynamic changes were recorded by electrocardiogram at different stages of I/R. Subsequently, reperfusion duration was used as a variable to directly compare the phenotypes of different myocardial injury degrees induced by 3 h, 6 h and 24 h reperfusion from myocardial infarct size, myocardial apoptosis, myocardial enzyme, and inflammatory cytokine levels.

Results: All mice subjected to myocardial I/R surgery showed obvious myocardial infarction, extensive myocardial apoptosis, dynamic changes in serum myocardial enzyme and inflammatory cytokines, at least for the first 24 h of reperfusion. The infarct size and apoptosis rates gradually increased with the extension of reperfusion time. The peaks of serum myocardial enzyme and inflammatory cytokines occurred at 6 h and 3 h of reperfusion, respectively. We also established I/R mice models with 30 and 60 mins of ischemia. After 21 days of remodeling, longer periods of ischemia increased the degree of fibrosis and reduced cardiac function.

Conclusions: In summary, we conclude that reperfusion durations of 3 h, 6 h, and 24 h induces different injury phenotypes in ischemia-reperfusion mouse model. At the same time, the ischemia duration before reperfusion also affects the degree of cardiac remodeling.

## INTRODUCTION

Myocardial infarction (MI) occurs when the blood supply to the heart is interrupted, and causes damage and possible death of the heart tissue [1]. Timely reperfusion (percutaneous coronary intervention or thrombolysis) is essential to salvage ischemic myocardium from the infarction, decrease in-hospital mortality, and improve the long-term outlook in survivors of the acute phase [2, 3]. Whereas, reperfusion itself enhances acute or chronic myocardial damage, known as myocardial ischemia-reperfusion injury (I/RI) [4]. Myocardial ischemia-reperfusion (I/R) is a complex pathophysiological event, when blood supply is restored after a period of ischemia, resulting in the production of free oxygen radicals, oxidative stress, excessive reactive oxygen species (ROS) generation [5], release of cytokines [6], inflammation [7], and endothelial cell dysfunction [8], eventually leading to cardiomyocyte apoptosis and death, accompanying with myocardial remodeling and decreased cardiac contractility and cardiac function [9]. Up to now, there is still no effective drug to prevent myocardial reperfusion injury. Therefore, understanding the molecular mechanism and the complex cellular events during myocardial ischemia-reperfusion injury has important clinical implications in the development of new therapies for this clinical set. Preclinical studies rely heavily upon animal models of human disease. A routine model of myocardial ischemia-reperfusion injury in mice is achieved through temporary ligation of the left anterior descending (LAD) artery followed by a period of reperfusion [10]. However, the uniformity in surgical procedures among different operators can hardly guarantee. In addition, the selection of the time window may bring limitations to the experimental results. Therefore, the accuracy and validity of the model are very important for both mechanistic studies and drug development. However, few studies focus on the direct comparison of the extent of pathological events resulting from different ischemia and reperfusion times.

Inflammatory damage has been shown to be a characteristic pathologic process in myocardial I/RI and it is also one of the reasons for the aggravation of myocardial injury and the enlargement of myocardial infarction area [11, 12]. Moreover, it has also been demonstrated that the inhibition of inflammation significantly reduced myocardial I/RI [13, 14]. Therefore, it is very important to clarify the rules of inflammatory response in myocardial I/RI, and timely suppression of the inflammatory response at a specific time to maintain the balance of inflammatory response. Excessive apoptosis has long been regarded as a facilitator for the pathological progression of myocardial I/RI and inhibition of apoptosis thereby becomes a worthwhile approach to attenuate myocardial I/RI [15, 16]. Hypoxia caused by MI, and oxidative stress and inflammation caused by reperfusion all lead to cardiomyocyte apoptosis. Therefore, it is necessary to fully understand the main stages and reason of myocardial apoptosis following MI, so as to select appropriate treatment methods and time. The over-deposition of extracellular matrix proteins following myocardial I/RI may lead to myocardial fibrosis, which may induce myocardial pathological remodeling and decrease cardiac function [17, 18]. *In vivo* studies in mice, the degree of myocardial fibrosis and scar area are important indexes to evaluate cardiac function after I/RI in mice.

In the present study, we evaluated the different response to different durations of ischemia and reperfusion in mice, and analyzed the effects of different ischemia and reperfusion times on myocardial injury, inflammatory response, myocardial apoptosis, myocardial fibrosis, and cardiac function. Our study may provide a clear approach to the construction of mice myocardial I/RI models for researchers, so that they can choose the appropriate timing, combination, and determine the schedule of end point of the experiment.

## RESULTS

Focusing on a mouse model of myocardial I/RI, a total of 70 mice were used in this study. Except for the deaths caused by technical accidents during I/R surgery and prolonged pathological cardiac remodeling, 21 mice were used for cardiac TTC staining analysis, 16 mice were sacrificed for collection of myocardial tissue and blood samples, and 15 mice were subjected to echocardiography to measure cardiac function and myocardial tissue section preparation for histopathology.

### Electrocardiography

**Figure 1:**
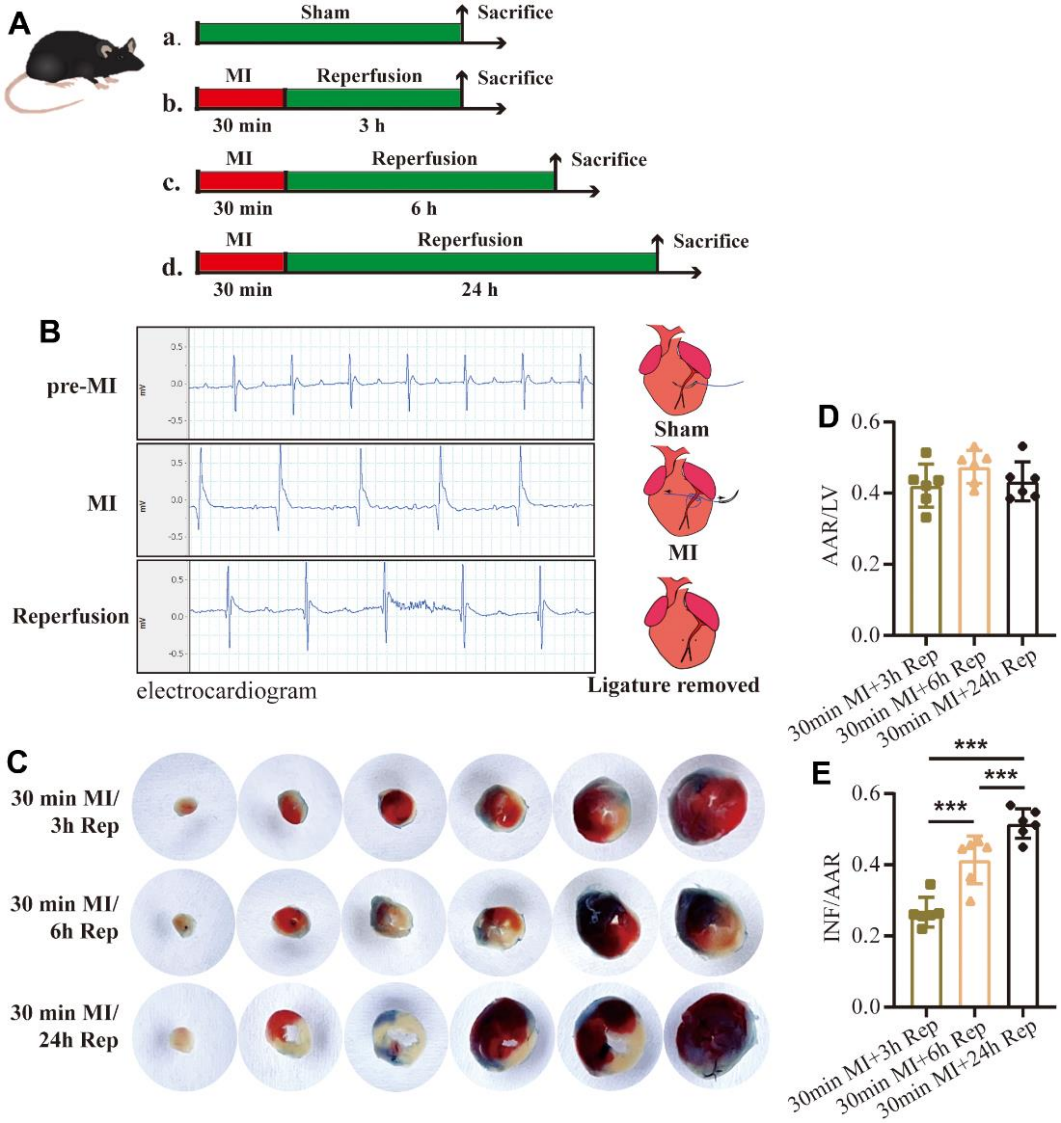
**Influence of duration of reperfusion time on infarct size.** (**A**) Flow chart of the experimental protocols. (**B**) Representative ECG tracings of each group in mice. (**C**) Representative images of 2,3,5-triphenyltetrazolium chloride (TTC) staining and quantification of the area at risk/left ventricular weight (**D**, AAR/LV) ratio and the infarct size/area at risk (**E**, INF/AAR) ratio (n = 6 : 6 : 6). MI=Myocardial infarction, Rep=reperfusion. Data are shown as mean ± SD. One-way ANOVA followed by Tukey post hoc test was used for statistical comparisons between multiple groups. ***P < 0.001.

The flow chart of the experimental protocols in the study is given in Figure 1A. Firstly, 3 groups of mice were subjected to 30 mins of left anterior descending coronary occlusion followed by 3, 6, and 24 h reperfusion, respectively. The establishment of the I/R model was first verified with ECG detection. As shown in Figure 1B, mice in the sham group showed normal ECG, while the significantly elevated ST segment of lead II ECG and T wave inversion were observed in LAD occlusion mice. In addition, the ECG showed a decreased ST segment and T wave after removing the ligature. Moreover, arrhythmia appeared in the early reperfusion phase, after which the heart rate gradually stabilizes. Therefore, the ECG results suggest the success of myocardial reperfusion model.

### Influence of duration of reperfusion time on infarct size

To evaluate the outcomes of different reperfusion times on myocardial infarction, we measured the ischemic area (area at risk, AAR) and the infarct (INF) area in each group by TTC and Evans blue double-staining (Figure 1C). There was no significant difference in AAR relative to left ventricular area (AAR/LV) ratio among groups, suggesting a good surgical uniformity among all groups (Figure 1D). Moreover, infarct area increased significantly with duration of reperfusion. Prolongation of reperfusion from 3 h to 6 h increased INF size from 27.97% ± 3.7% to 42.64% ± 6.1%, and 52.85% ± 3.7% in 24 h of reperfusion group, respectively (Figure 1E). Therefore, new irreversible myocardial injury in the mice can still occur after 3 h of reperfusion following myocardial ischemia for 30 mins and cumulative myocardial injury from 6 to 24 h after reperfusion can result in a marked extension of the size of myocardial infarct.

### Detection of apoptotic cell after ischemia and reperfusion

**Figure 2:**
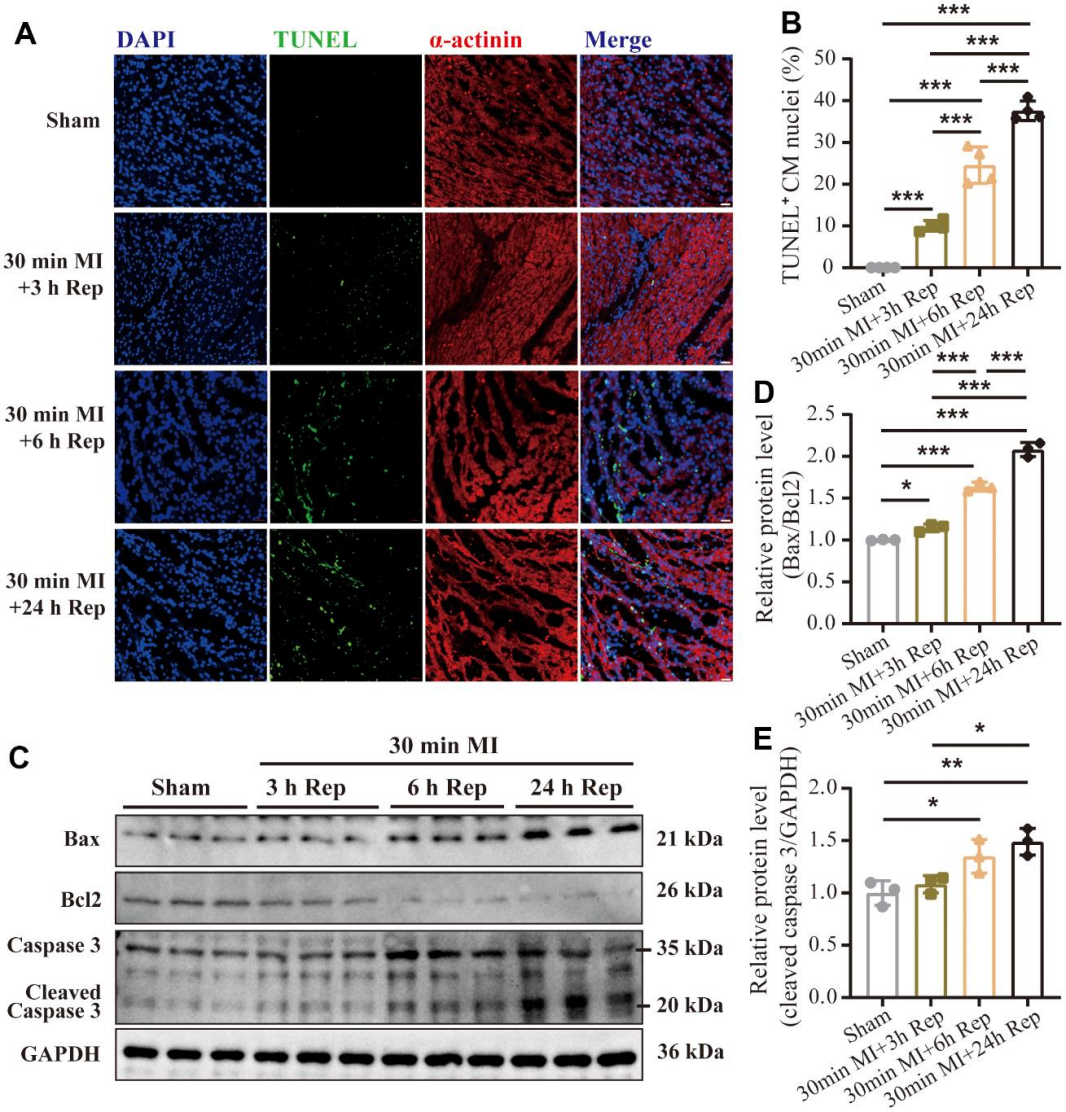
**Progression of apoptotic cell after ischemia and reperfusion.** (**A**) Representative images of immunofluorescence staining and (**B**) quantification of the TUNEL positive cardiomyocytes in mouse hearts treated as indicated (n = 4 : 4 : 4 : 4) Scale bar: 100 μm. (**C**) Representative western blot and (**D**, **E**) statistical data of myocardium apoptosis by detection of Bax, Bcl2, and Cleaved-Caspase 3 expression levels in mouse hearts treated as indicated (n = 3 : 3 : 3 : 3). MI=Myocardial infarction, Rep=reperfusion. Data are shown as mean ± SD. One-way ANOVA followed by Tukey post hoc test was used for statistical comparisons between multiple groups. *P < 0.05; **P < 0.01; ***P < 0.001.

Detection of myocardial apoptosis by TUNEL staining and western blot analyses for apoptosis markers were performed on the myocardial tissue. As shown in Figure 2A, there were almost no TUNEL-positive cardiomyocytes were detected in myocardial tissues from the sham group. After 3 h reperfusion, the percentage of TUNEL-positive cells nuclei in the zone of the previously ischemic area increased to 9.99% ± 1.1% (Figure 2B). Moreover, the apoptotic cell number gradually increased in 6 h (24.56% ± 3.8%) and 24 h (37.58% ± 2.0%) groups following reperfusion time extension. Furthermore, the western blot results also confirmed that the expression of pro-apoptotic proteins Bax and cleaved caspase-3 were increased with the extension of reperfusion time (Figure 2C–2E). The above results suggest the dynamic progression of apoptosis and infarct extension during the late phase of reperfusion.

### Effect of reperfusion duration on myocardial enzyme release

**Figure 3:**
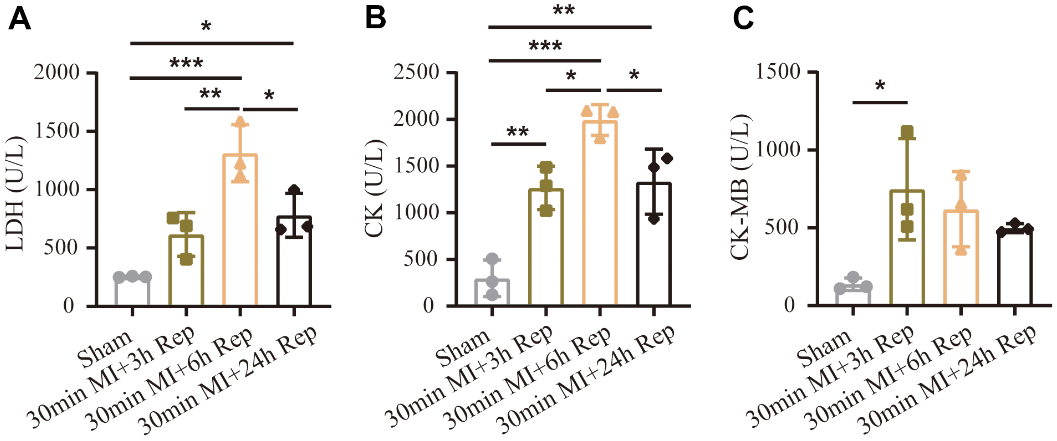
**Effect of reperfusion duration on myocardial enzyme release.** (**A**) Myocardial enzyme concentrations of LDH. (**B**) Myocardial enzyme concentrations of CK. (**C**) Myocardial enzyme concentrations of CK-MB (n = 3 : 3 : 3 : 3). MI=Myocardial infarction, Rep=reperfusion. Data are shown as mean ± SD. One-way ANOVA followed by Tukey post hoc test was used for statistical comparisons between multiple groups. *P < 0.05; **P < 0.01; ***P < 0.001.

In addition to infarct and TUNEL staining, we evaluated serum myocardial enzyme as marker of ischemic myocardial injury. The variations in enzyme levels following reperfusion are shown in Figure 3. In fact, the serum levels of lactate dehydrogenase (LDH), creatine kinase (CK) and creatine kinase-MB (CK-MB) were significantly up-regulated during reperfusion, among which LDH and CK reached to the peak value at 6 h, whereas CK-MB showed highest value 3 h after reperfusion. The dynamic changes of release of these myocardial enzymes are of particular interest during early reperfusion after myocardial ischemia.

### Cytokine concentrations following reperfusion

**Figure 4:**
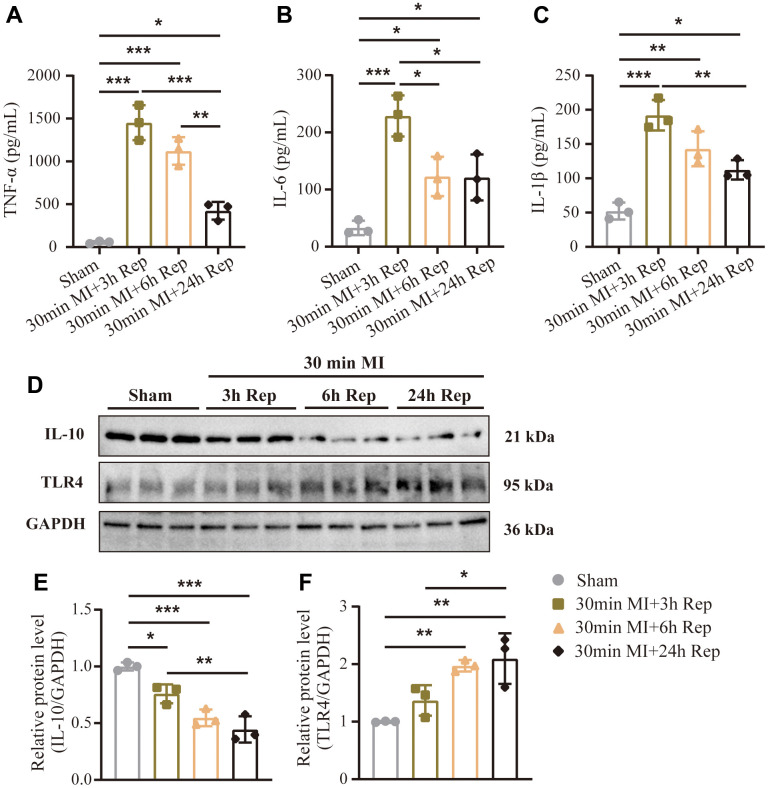
**Dynamic changes in serum inflammatory cytokines following reperfusion.** (**A**) Serum inflammatory cytokines concentrations of TNF-α. (**B**) Serum inflammatory cytokines concentrations of IL-6. (**C**) Serum inflammatory cytokines concentrations of IL-1β (n = 3 : 3 : 3 : 3). (**D**) Representative western blot and (**E**, **F**) statistical data of myocardium inflammation by detection of IL-10 and TLR4 expression levels in mouse hearts treated as indicated (n = 3 : 3 : 3 : 3). MI=Myocardial infarction, Rep=reperfusion. Data are shown as mean ± SD. One-way ANOVA followed by Tukey post hoc test was used for statistical comparisons between multiple groups. *P < 0.05; **P < 0.01; ***P < 0.001.

Based on the results above, we boldly speculate that different reperfusion time may directly affect the severity of ischemia-reperfusion injury and inflammation. Variations in serum cytokine levels following reperfusion are shown in Figure 4A–4C for the groups as a whole. Tumor necrosis factor alpha (TNF-α), *interleukin* 6 (IL-6) and *interleukin*-1β (IL-1β) levels constantly rose at the beginning of reperfusion and reached a maximum concentration after 3 h. The content of inflammatory factors decreased gradually with the extension of reperfusion time, but still remained higher than the normal level at least after 24 h of reperfusion. Furthermore, the western blot results also confirmed that the expression of anti-inflammatory cytokines IL-10 in myocardial tissue was decreased with the extension of reperfusion time, while the expression level of Toll-like receptor 4 (TLR4) was on the contrary (Figure 4D–4F).

### Effect of different durations of ischemia on cardiac remodeling after reperfusion

**Figure 5:**
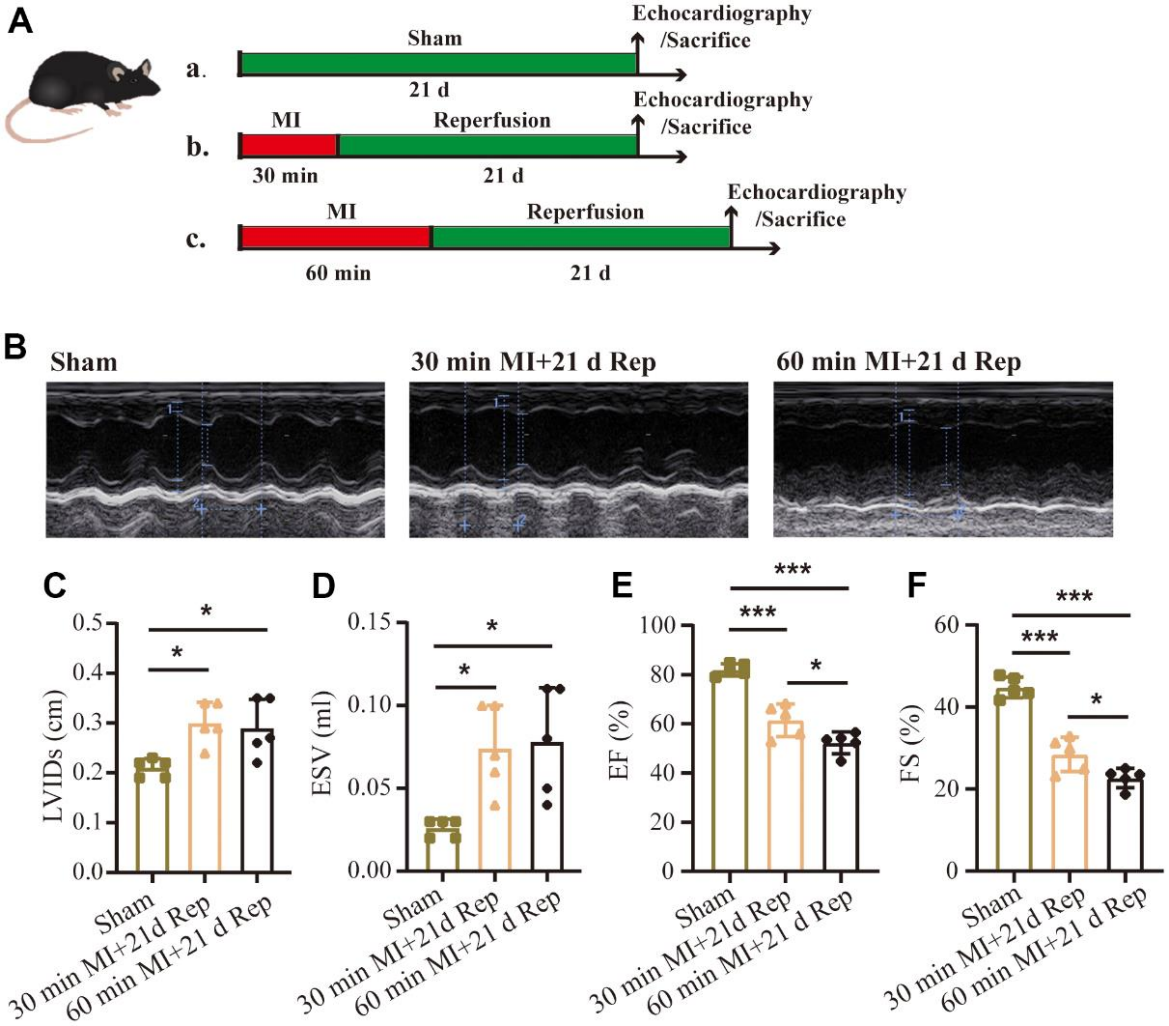
**Effect of different durations of ischemia on heart failure after reperfusion.** (**A**) Flow chart of the experimental protocols. (**B**) Representative echocardiographic images and statistical data of left ventricular internal dimension in systole (**C**, LVIDs), end-systolic volume (**D**, ESV), left ventricular ejection fraction (**E**, EF%), fractional shortening (**F**, FS%) of mice (n = 5 : 5 : 5 : 5). MI=Myocardial infarction, Rep=reperfusion. Data are shown as mean ± SD. One-way ANOVA followed by Tukey post hoc test was used for statistical comparisons between multiple groups. *P < 0.05; ***P < 0.001.

Myocardial ischemia-reperfusion injury leads to aggravated pathological myocardial remodeling and heart failure. However, in mice models, different durations of ischemia before reperfusion may also result in distinct phenotypes. As shown in Figure 5A, 2 groups of mice were subjected to 30 mins and 60 mins of ischemia via ligation of the left anterior descending coronary artery, followed by reperfusion. Heart function of the mice was evaluated by echocardiography at 21 days after surgery (Figure 5B). Compared to the sham operation group, 30 and 60 mins ischemia followed by reperfusion caused significantly increased left ventricular internal dimension in systole, LVIDs (Figure 5C), end-systolic volume, ESV (Figure 5D) and decreased ejection fraction, EF% (Figure 5E), fractional shortening, FS% (Figure 5F). However, under the same reperfusion time and remodeling conditions, early ischemia for 60 mins further deteriorated cardiac function (Figure 5E, 5F).

**Figure 6:**
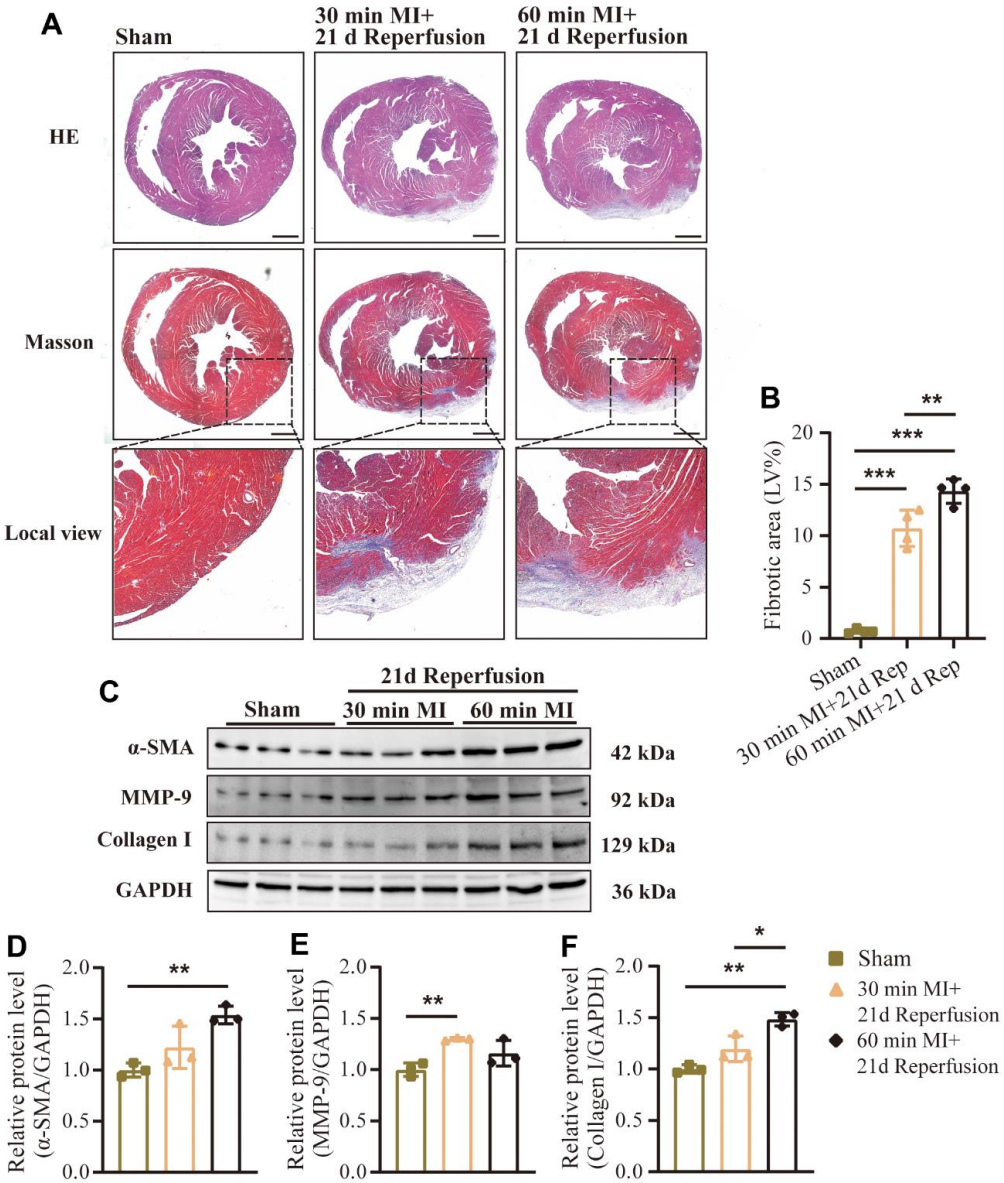
**Effect of different durations of ischemia on cardiac remodeling after reperfusion.** (**A**) Representative images of HE and Masson’s trichrome staining and (**B**) quantification of fibrotic area (%) in mouse hearts treated as indicated (n = 4 : 4 : 4 : 4). Scale bar: 500 μm. (**C**) Representative western blot and (**D**–**F**) statistical data of myocardial fibrosis by detection of (**D**) α-SMA, (**E**) MMP-9, and (**F**) collagen I expression levels in mouse hearts treated as indicated (n = 3 : 3 : 3 : 3). MI=Myocardial infarction, Rep=reperfusion. Data are shown as mean ± SD. One-way ANOVA followed by Tukey post hoc test was used for statistical comparisons between multiple groups. *P < 0.05; **P < 0.01.

Subsequently, myocardial tissue sections were prepared in order to analyze the pathological changes through H&E and Masson staining. As shown in Figure 6A, there was a visible scar area in the anterior wall of the left ventricle in I/RI mice and the scar area in the 60 mins ischemia group was slightly larger than that in the 30 mins ischemia group. Moreover, there was obvious collagen deposition in the scar area, and the fibrosis area in the 60 mins ischemia group (14.33 ± 1.0%) was significantly larger than that in the 30 mins ischemia group (10.71% ± 1.5%) (Figure 6B). This distinct phenotype was also confirmed by western blotting of fibrotic proteins (α-SMA, MMP-9 and Collagen I) in ischemic lesions of the myocardial tissue (Figure 6C–6F).

## DISCUSSION

Mouse models of myocardial ischemia-reperfusion injury are widely used to investigate the cascade of events occurring after myocardial ischemia/reperfusion injury. However, due to the difficulty and instability of the surgery, some experimental results are difficult to reproduce. This study describes methodological details of this model and investigated the impact of ischemia and reperfusion duration on distinct phenotypes in mice to provide a clear approach to the construction of mice myocardial I/RI models for researchers, so that they can choose the appropriate timing, combination, and determine the schedule of end point of the experiment.

Myocardial ischemia-reperfusion can be induced by transient LAD ligation and the sufficient occlusion may be verified by blanching of the cardiac tissue distal to the ligation or by an ST-elevation in the electrocardiography. In the reported literature, typical ligature usually lasts between 20 and 60 mins [19–23]. However, less than 30 mins of LAD ligation may not induce ischemia-induced myocardial injury, whereas 60-90 mins of ligation may result in complete infarction of the ischemic area and irreversible myocardial cell death [24]. Therefore, 30-60 mins may be the appropriate duration for ischemia, and the period of ischemia and reperfusion varies from several hours to several weeks [19, 24, 25], depending on the purpose of each study.

In the present study, a mouse model of myocardial ischemia reperfusion was established by ligation and reperfusion of the left anterior descending coronary artery, and the dynamic changes during myocardial ischemia and reperfusion were recorded by electrocardiogram at different stages of I/R (basic state, ischemia state and reperfusion state). Subsequently, reperfusion duration was used as a variable to directly compare the phenotypes of different myocardial injury degrees induced by 3 h, 6 h and 24 h reperfusion from four aspects: myocardial infarct size, myocardial apoptosis, myocardial enzyme levels and inflammatory cytokines levels. All mice subjected to I/R surgery showed obvious myocardial infarction and extensive myocardial apoptosis, and the infarct size and apoptosis rates gradually increased with the extension of reperfusion time, at least for the first 24 h of reperfusion. The enlargement of infarct size with increasing duration of reperfusion after 30 mins of MI as seen in this study may, at least in part, be due to increased apoptotic cell death.

During myocardial ischemia, the integrity of the cell membranes is damaged, causing myocardial enzyme to be released into the peripheral blood [26, 27]. The elevated levels of CK, CK-MB, and LDH could reflect the changes in myocardial injury degree in different periods [28]. Therefore, we also measured the serum levels of myocardial enzyme to determine the dynamic pattern of myocardial enzyme during reperfusion. In fact, myocardial enzyme levels are upregulated at the beginning of myocardial ischemia or reperfusion, but different enzyme were differed in the level and time of occurrence of peak enzyme activity. During the last decades, activity measurements of myocardial enzyme, and especially the isoenzymes of CK and LDH, have become the final arbiters by which myocardial damage is diagnosed or excluded [26]. There are differences in the level of myocardial enzyme in animal experiments and clinical trials. However, in basic research, it is also extremely important to determine the dynamic level of myocardial enzyme and select the appropriate detection time point.

A number of studies supported that the inflammatory response is one of the major mechanisms involved and plays a pivotal role in the pathogenesis of myocardial I/RI [29, 30]. It has also been demonstrated that the inhibition of inflammation significantly reduced myocardial I/RI [31]. It was reported that the pathologic process of myocardial I/RI was an acute inflammatory reaction, which can then cause multiple pathological changes, including acute inflammatory cascade response and apoptosis. When reperfusion injury occurs, the expression of pro-inflammatory factors, adhesive molecules, cytokines, and chemokines can also be up-regulated and then induce cell apoptosis. Our results also suggest that serum TNF-α, IL-6 and IL-1β levels were immediately upregulated at the beginning of reperfusion and subsequently decreased, but the levels of these inflammatory cytokines still remained higher than those in the sham group until 24 h. Many studies suggest that TLR4 signaling pathway plays an important role in the transmission of inflammatory signals [32, 33]. A large number of pro-inflammatory factors, chemokines and adhesion factors interact with TLR4 to promote the development of the disease [34]. Western blotting showed that TLR4 expression was up-regulated and anti-inflammatory IL-10 expression was down-regulated during reperfusion in a time-dependent manner. Our results suggest that the degree of inflammatory response during reperfusion is closely related to the duration of reperfusion, and the overheated inflammatory response may be the main cause of increased apoptosis of cardiomyocytes in the late reperfusion period.

Among the recognized mechanisms of myocardial ischemia-reperfusion injury, oxidative stress also has become an area of increasing interest and research focus [35]. Imbalance between the production of oxygen free radicals and the capacity of the antioxidant systems of the myocardium may lead to myocardial injury and dysfunction [36]. Several recent evidence has led to the attempt to affect the redox status of the myocardium to restore the necessary biochemical balance. For example, it has been shown that H_2_S therapy enhances phosphorylation and nuclear localization of nuclear factor erythroid 2-related factor 2 (NRF2), leading to the expression of NRF2-targeted downstream anti-oxidative stress genes, thus protecting cardiac tissue against I/R injury [37]. Moreover, antioxidants, free radical scavengers and traditional Chinese medicine can also protect against myocardial damage in ischemic heart disease [38–42]. Therefore, targeting oxidative related signaling pathways offers a strategic approach in myocardial IR/I therapeutic, and the Future work should carefully evaluate potential associations between different ischemia and reperfusion times and the level of Markers of oxidative stress injury like reactive oxygen species (ROS), Heme Oxygenase 1 (HO-1), super oxide dismutase (SOD) and NRF2.

The degree and time of myocardial remodeling after injury in mice depended on the time of LAD ligation and the use of reperfusion protocol [43]. Because of a high variety of experimental protocols, reported data from different laboratories cannot be compared directly. Therefore, we established myocardial I/R mice models with 30 and 60 mins of ischemia, and evaluated the degree of cardiac remodeling after 21 days. Interestingly, longer periods ischemia in the prior period increased the degree of fibrosis in the scar area and further reduced cardiac function. This phenomenon may be due to the increased apoptosis of cardiomyocytes caused by prolonged ischemia in the early stage, which eventually leads to the increased lesion area. In addition, hypoxia-induced overactivation of fibroblasts may promote scar formation, thereby increase fibrosis and collagen metabolism disorders.

The finding that myocardial infarction size increases with the reperfusion time extension is controversial, but several previously published studies are in accordance with this finding [44, 45]. Our results support the concept of reperfusion injury indicating that reperfusion of ischemic tissue introduces additional lethal injury. In summary, based on the results, we conclude that reperfusion durations of 3 h, 6 h, and 24 h induces different injury phenotypes in ischemia-reperfusion model mice. At the same time, the ischemia duration before reperfusion also affects the degree of long-term cardiac remodeling. Our study, for the first time, systematically evaluated the commonly used parameters for I/RI under different ischemia and reperfusion time points, and provided a clear approach to the construction of mice myocardial I/RI models for researchers. It may serve as a useful guide in the field for designing mice I/RI studies.

## MATERIALS AND METHODS

### Animals

All male C57BL/6 J mice aged 8–10 weeks were purchased from GemPharmatech Co. Ltd., (Chengdu, China) and raised at the specific pathogen-free laboratory animal facility of West China Hospital, Sichuan University (Chengdu, China). All efforts were made to minimize suffering in animals. At the end of the experiment, mice were anaesthetized with 2% isoflurane and euthanized by cervical dislocation.

### Myocardial I/R surgery

All mice were anaesthetized in the chamber at 2% isoflurane, under sterile conditions. Myocardial ischemia-reperfusion injury was induced by ligation of the left anterior descending artery (LAD) by a 7-0 silk suture for 30 mins or 60 mins followed by reperfusion for 3 h, 6 h and 24 h (acute I/R injury) or 21 days (I/R remodeling). Sham-operated mice were treated with the same surgery without tying the left anterior descending coronary artery. Electrocardiography was performed to evaluate the successful generation of the MI model. After that, mice were sacrificed at various time points, and myocardial tissue was collected for further analysis.

### Electrocardiogram

Electrocardiogram (ECG) was performed immediately after MI surgery. All mice were anaesthetized with isoflurane, carefully positioned on the ECG recording platform. Surface lead II ECG was obtained. After electrode setup and system adjustment were accomplished, the next 1 min of ECG recordings were analyzed by LabChart 8.2.3 (AD Instruments, New South Wales, Australia). In this experiment, three stages of ECG were recorded: before operation, myocardial ischemia and reperfusion.

### TTC staining

The infarct size was determined by TTC staining. 3 h, 6 h and 24 h after reperfusion, the LAD was re-ligated at the same location, 1 mL Evan’s Blue (Sigma) was injected via the aorta to visualize the area at risk (AAR). Then the hearts were immediately stored at -20° C for 30 min followed by slicing 2 mm thick from the apex of the heart to the aorta. The sections were then stained with 1% TTC for 20 min at 37° C. To assess surgery homogeneity, area at risk/left ventricle weight (AAR/LV) was calculated. The infarct size/area at risk (INF/AAR) was calculated to evaluate the myocardial infarction severity.

### TUNEL staining

TUNEL BrightRed Apoptosis Detection Kit (Vazyme, A113-01) was used to perform TUNEL staining on 5 μm frozen heart sections according to the manufacturer’s protocol after α-Actinin-Cy3 antibody. All sections and glass bottom plates were examined by confocal microscope (ECLIPSE Ti A1, Nikon, Tokyo, Japan). TUNEL positive and α-actinin positive nuclei were counted for cardiomyocyte TUNEL positive.

### Western blotting

Myocardial tissue and cell suspension was lysed by RIPA lysis buffer (MB-030-0050, Multi Sciences Biotech, Hangzhou, China). BCA Protein Assay Kit (23225, Thermo Fisher, USA) was adopted to determine protein concentrations. After equal quantities, total proteins were separated in SDS-PAGE gel (4-20%, ACE Biotechnology, Xiangtan, China), and the bands were transferred onto PVDF membranes (03010040001, Merck, USA). After blocked with 5% skimmed milk and washed with tris-buffered saline containing 0.1% tween-20 (TBST), protein bands were blotted with primary antibodies at 4° C overnight. After washing with TBST, protein bands were blotted with HRP conjugated secondary antibody and monitored using ECL buffer (32209, Thermo Fisher). Quantifications of western blotting were measured with Fusioncapt advance software. Antibody information: Bax (HUABIO, USA, ET1603-34, 1:1000), Bcl2 (HUABIO, ET1702-53, 1:1000), Cleaved-caspase3 (HUABIO, ET1608-64, 1:1000), IL-10 (Servicebio, Wuhan, China, GB11108, 1:1000), TLR4 (Servicebio, GB11519, 1:1000), α-SMA (Servicebio, GB111364, 1:1000), MMP-9 (Abcam, Cambridge, UK, ab76003, 1:1000), Collagen I (Abcam, ab34710, 1:1000), GAPDH (CST, USA, 5174S, 1:1000). HRP-conjugated secondary antibody (HUABIO, HA1001, and HUABIO, HA1006, 1:50000).

### Enzyme-linked immunosorbent assay (ELISA)

ELISA was performed to measure the levels of myocardial enzyme and inflammatory cytokine in mice serum. Quantitative measurement of LDH, CK, CK-MB, IL-6, TNF-α and IL-1β was performed in serum supernatants using LDH ELISA Kit (Rayto, S03034), CK ELISA Kit (Rayto, S03024), CK-MB ELISA Kit (Changchun Huili, Changchun, China, C060), IL-6 ELISA Kit (Thermo Fisher, 88-7064-88), TNF-α ELISA Kit (Thermo Fisher, 88-7324-88), and IL-1β ELISA Kit (88-7013-22) respectively, according to manufacturer’s manuals.

### Echocardiography

All mice underwent transthoracic echocardiography under 1.5% isoflurane anaesthesia. The cardiac function (left ventricular ejection fraction, LV EF and fractional shortening, FS) were detected by Vivid7 Dimension system (GE, USA) with a 30 MHz central frequency scan head and measured from M-mode images taken from the parasternal short-axis view at papillary muscle level.

### Histological analysis

Mice myocardial tissue was fixed in 4% paraformaldehyde followed by embedded with paraffin. Pieces of heart embedded in paraffin were cut into 5 μm sections and mounted on glass slides. After dewaxing and dehydrating with xylene and ethanol, the sections were stained successively with hematoxylin and eosin staining, iron hematoxylin staining, ponceau acid magenta, phosphomolybdate staining, and aniline blue dyeing. Images were acquired using a bright field microscope (Olympus, Tokyo, Japan). Myocardial infarct scar area was analyzed by ImageJ.

### Statistical analysis

Data from the mouse and cell model were expressed as mean ± SD. Significant differences were assessed by One-way ANOVA followed by Tukey post hoc test which was used for statistical comparisons between multiple groups. A value of *P* < 0.05 was considered to be statistically different. Analyses were performed using GraphPad Prism 8.
